# Serological Assays to Measure Rabies Antibody Response in Equine Serum Samples

**DOI:** 10.3390/v18010108

**Published:** 2026-01-14

**Authors:** Nisha Beniwal, Banwari Lal, Sushma Mithina, Chandan Kumar Verma, Satendra Kumar, Vikas Phagna, Kamini Jakhar, Sudipta Sonar, Vishal Gupta, Rita Singh, Niraj Kumar, Chee Wah Tan, Riyesh Thachamvally, Harisankar Singha, Kripa Murzello, Aldon Fernandes, Lin-Fa Wang, Sankar Bhattacharyya, Shailendra Mani

**Affiliations:** 1 Biotechnology Research and Innovation Council Translational Health Science and Technology Institute, Faridabad 120001, Haryana, Indiachandanverma@thsti.res.in (C.K.V.); ritasingh@thsti.res.in (R.S.); nkumar@thsti.res.in (N.K.); 2 Programme in Emerging Infectious Diseases, Duke-NUS Medical School, 8 College Road, Singapore 169857, Singapore; 3 ICAR-National Research Centre on Equines, Sirsa Road, Hisar 125001, Haryana, India; 4 Bharat Serums and Vaccines Limited, Navi Mumbai 400708, Maharashtra, India

**Keywords:** rabies virus, rabies pseudovirus, antibody response, virus neutralization, luminex bead-based immunoassay

## Abstract

Rabies is a neglected tropical zoonotic disease caused by rabies-virus (RV) infection and is responsible for almost 60,000 annual deaths globally, largely affecting the socio-economically disadvantaged population. Although fatality is preventable by immunization either before or after exposure with therapeutic antibodies, the high cost of prophylaxis or treatment limits their accessibility for the affected population. However, due to the almost 100% fatality rate in symptomatic individuals, almost 29 million annual vaccinations are performed, imposing high financial burden. Human transmission occurs principally through bites from infected dogs and although multiple mammalian species are permissive to RV, transmission from them or from symptomatic humans is rare. To overcome the limitations posed by the requirement of biosafety level-3 (BSL-3) containment for live virus culture, we established a replication-deficient vesicular stomatitis virus (VSV) pseudovirus expressing the Rabies-G (RV-G) protein and a multiplexed Luminex immunoassay for quantifying anti-rabies antibodies in equine sera. The purified pseudovirus exhibited robust luciferase activity and was able to infect multiple mammalian cell lines, although with variable efficiency. Using hyper-immunized equine serum, we observed a strong correlation (ρ > 0.9, *p* < 0.001) between binding antibody titers measured by the Luminex assay with neutralizing antibody titers determined using the pseudovirus-based neutralization assay. These assays provide a safe, quantitative, and BSL-2-compatible platform for rabies serological evaluation and vaccine testing.

## 1. Introduction

Rabies is a highly fatal encephalitic disease affecting both animals and humans, caused by the rabies virus (RV), a member of the Lyssavirus genus within the Rhabdoviridae family, causing nearly 60,000 deaths annually worldwide [1]. Human infection primarily occurs through bites or scratches from rabid animals, especially dogs (99%), while transmission between humans is exceedingly rare. Although deadly, it can be effectively prevented with the use of a post-exposure prophylaxis (PEP) regimen, that includes prompt vaccination, administration of rabies immunoglobulins, and proper wound treatment [2,3]. Most rabies cases occur in Asia and Africa, particularly across Southeast Asian countries such as Bangladesh, Bhutan, the Democratic People’s Republic of Korea, India, Indonesia, Myanmar, Nepal, Sri Lanka, and Thailand [4]. India, with its vast population and diverse animal species, faces substantial challenges in rabies control and management. Understanding the rabies epidemiology and the advancements in vaccine development is crucial for effective disease management and prevention strategies [5].

RV is an RNA virus characterized by a unique “bullet” morphology with an approximately 12 kb genome that encodes five proteins: polymerase (L), matrix protein (M), glycoprotein (G), phosphoprotein (P), and nucleoprotein (N). Rhabdoviruses are characterized by their helical ribonucleoprotein core (RNP) which encapsulates the genomic RNA, surrounded by an envelope. The surface of the virus is tightly packed with about 400 trimeric spikes formed by the glycoprotein (G), a type I transmembrane protein. The Rabies-G protein is important for host cell entry by mediating the interaction of virion particles with the receptor and its immunodominance, inducing neutralizing antibodies make it the most significant immunogen in vaccine formulation. As a result, G protein is critical for establishing antigenicity and host range [6].

In India, detection of rabies infection and treatment for the symptoms are generally handled by a combination of government hospitals, research institutions, and specialized centers. However, despite these efforts, there remains a significant gap in safe and accessible systems for evaluating vaccine-induced immunity and PEP antiviral efficacy. Accessibility and restriction to handle the RV in higher containment biosafety level 3 (BSL-3) make it more difficult for laboratories to evaluate efficacy and neutralizing antibodies, which impedes the development of antivirals and vaccines. Additionally, there is a significant demand for vaccine candidates, yet the supply of these vaccines is often constrained due to several factors, including insufficient evaluation of vaccine efficacy and the challenges associated with conducting trials in a BSL3 environment. The stringent requirements of BSL3 facilities, which are designed to handle potentially lethal agents, restrict the number of locations where vaccine development can occur. To address these challenges, Pseudovirus-based systems offer a safe BSL-2 surrogate to study viral entry, neutralization, and vaccine efficacy. These surrogate models reduce biosafety constraints, enhance research capacity, and have the potential to accelerate the development of effective rabies vaccines [7].

To overcome this necessary safety restriction, we intend to establish a serological assay in which viral entry, neutralization, and vaccine efficacy can be evaluated within a BSL-2 facility, making the platform safer and more convenient to use. We have established and characterized a Rabies-G-containing vesicular stomatitis virus (VSV)-Luciferase (Luc) pseudovirus system, engineered to carry reporter gene encoding luciferase protein [8,9]. Characterization and quantitative analyses on these pseudoviruses are easier than on wild types as the number of pseudovirus-infected cells is directly proportional to reporter gene activity. Therefore, this study aimed to establish the rabies-pseudovirus system and evaluated its efficacy using neutralization assay for the quantification of rabies-neutralizing antibodies present in the serum of immunized equine samples.

## 2. Materials and Methods

### 2.1. Cells

HEK293T (ATCC), BHK-2l (ATCC), VERO E6 (ATCC), A549 cell lines were used in this study to evaluate the propagation and expression of rabies-pseudovirus. All the cells were maintained in high glucose DMEM (HiMedia, cat no.-AL007A) and supplemented with 10% fetal bovine serum (FBS, Gibco Cat no.-RM1112), penicillin/streptomycin (100 IU/mL/100 μg/mL, HiMedia Cat no.-A002), and non-essential amino acids (HiMedia Cat no.-ACL006) in a 5% CO_2_ environment at 37 °C and passaged every alternate day. All the experiments were conducted under strict bio-containment (BSL-2) using standard operating procedures.

### 2.2. Plasmids

RV G plasmid construct [*RABVgp4* Rabies virus (strain SAD B19)] was procured from Addgene (Catalogue number—15785). The plasmid was constructed by inserting Rabies G gene into the pcDNA3.1 vector at the *BamH*I and *Sal*I restriction sites and ampicillin was used as a selector marker.

### 2.3. Serum Samples

The anti-rabies equine serum samples were obtained from Bharat Serum Ltd. In addition, equine serum samples were acquired from ICAR-National Research Center on Equines, Hisar, Haryana, India.

### 2.4. Generation of a Pseudo-Typed Virus-VSV-Rabies-G-Luc

**Figure 1:**
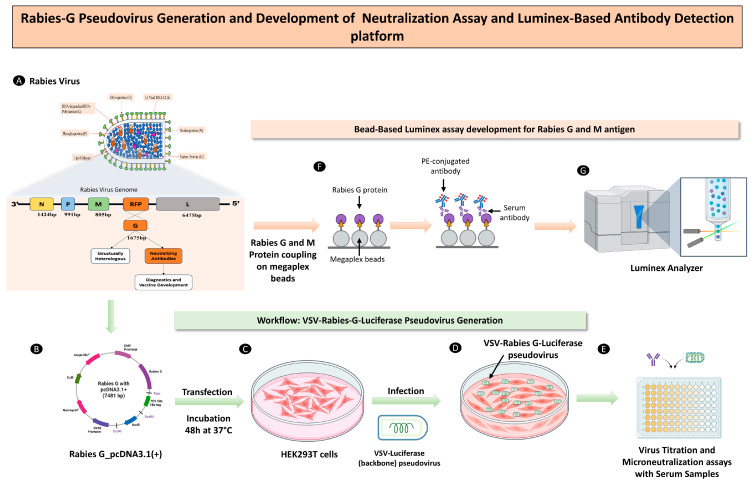
Schematic representation of VSV-Rabies-G-Luc pseudovirus generation, neutralization assay development, and luminex-based antibody detection platform. (**A**) The structure of the Rabies virus is depicting its bullet-shaped morphology, surface glycoprotein (G), and internal components including the nucleoprotein (N), phosphoprotein (P), matrix protein (M), and large RNA polymerase (L), which are all encoded by its single-stranded negative-sense RNA genome. (**B**) A pcDNA3.1 expression plasmid containing the rabies glycoprotein (G) gene is constructed for protein expression. (**C**) HEK293T cells are transfected with the pcDNA3.1-Rabies-G plasmid using poly-ethylenimine. After transfection, cells are incubated for 48 h to allow optimal expression of the Rabies-G protein on the cell surface. (**D**) Transfected cells are infected with ΔG-VSV-Luc (VSV lacking its glycoprotein), enabling incorporation of Rabies-G into VSV particles to generate replication-deficient VSV-Rabies-G pseudoviruses. Supernatant is harvested and clarified for downstream applications. (**E**) Virus titration curve was used to determine the optimal viral input for the assay. Neutralization assay is performed with VSV-Rabies-G pseudovirus and serum samples, and infection is quantified via luciferase readout after 24 h. Neutralizing antibody activity is assessed based on the reduction in luciferase signal. (**F**) Luminex assay development: Rabies-G and M protein antigens are covalently coupled to carboxylated magnetic beads using EDC/Sulfo-NHS chemistry. (**G**) Antigen-coated beads are incubated with serum samples, followed by detection using PE-conjugated anti-human IgG antibodies. The bead-associated fluorescence is measured using a Luminex analyzer to quantify antigen-specific IgG responses.

VSV pseudo-typed with Rabies G was produced by transfecting HEK293T cells at 80–90% confluency in serum-free DMEM with Rabies G-cloned plasmid DNA (pCDNA3.1), using PEI (Polysciences, Warrington, PA, USA, Cat no.-24765-1) at a 1:3 ratio. The culture medium was changed 4–6 h after transfection to media containing 2% FBS, and the cells were further incubated at 37 °C in a CO_2_ incubator for 48 h. Subsequently, the cells were infected with ΔG-VSVwith Luc backbone (gifted by Prof. Linfa Wang, Duke NUS Medical School, Singapore) at a multiplicity of infection (MOI) of one and the culture supernatant was collected 24 h post-infection, producing VSV-Rabies-G-Luc pseudovirus as shown in Figure 1. The supernatant was centrifuged at 2000 rpm for 5 min to remove cell debris, filtered through a 0.45 μm filter, and stored at −80 °C for future use.

### 2.5. Rabies-G Protein Validation by Western Blotting

To assess the expression of the Rabies-G protein after the transient-transfection of the Rabies-G-pcDNA3.1 plasmid into HEK293T cells, immunoblotting was performed. Briefly, cellular proteins were extracted using RIPA lysis buffer and separated on a 12% SDS-PAGE gel. The proteins were then transferred onto a PVDF membrane. The membrane was incubated overnight at 4 °C with primary antibodies against Rabies-G (Immunized Equine serum, 1:100) and GAPDH (G Biosciences, Cat no.-M1000110 (1:1000)). The membrane was washed with washing buffer thrice followed by incubation with HRP-tagged anti-rabbit secondary antibody (Cat no.-ITSAH134 (1:1000)) against Rabies-G antibodies and HRP-tagged anti-mouse secondary antibody (Cat no.-ITSAH131 (1:1000)) against GAPDH for 2 h at room temperature. Following additional washes, the protein bands were detected using an ECL substrate (Western blotting Luminol Reagent: sc-2048, Santa Cruz Biotech, Dallas, TX, USA) and visualized using a gel-doc (Bio-Rad, ChemiDoc^TM^ MP imaging system, Hercules, CA, USA).

### 2.6. Optimization of VSV-Rabies-G-Luc Pseudovirus Infection on Different Cell Lines

HEK293T, BHK-21, VERO E6, and A549 were seeded at 20,000 cells per well in a 96-well plate. The cells were infected directly with pseudovirus (6.25 µL) (VSV-Rabies-G-Luc) for 2 h. Afterward, the cells were washed with serum-free media, and 2% FBS-DMEM was added. The plates were incubated for 24 h, after which 30 μL of the culture medium was removed and replaced with an equal volume of luciferase substrate (britelite plus Reporter Gene Assay System, Revvity). The contents were mixed thoroughly and incubated for 2 min. Luciferase activity was then measured at a wavelength of 560 nm using the illuminometer (Fluoroskan Ascent, Thermo Fisher Scientific, Waltham, MA, USA).

### 2.7. VSV-Rabies-G-Luc Pseudovirus Titration

Two-fold serial dilutions of VSV-Rabies-G Luc in DMEM supplemented with 2% FBS were added to BHK-21 cellsseeded in 96-well culture plates. Luciferase activity in each well was evaluated after an incubation period of 20–24 h using illuminometer as described above. The titer of VSV-Rabies-G Luc was determined by finding the maximum dilution that produced positive bioluminescence determined as the relative light unit (RLU) and expressing it as infectious units (IUs).

### 2.8. Serum Neutralization Test with VSV-Rabies-G-Luc

The equine sera were diluted in a series of two-fold dilutions and mixed with an equal volume of VSV-Rabies-G Luc pseudovirus (50 µL) containing 6.25 µL (pseudovirus stock) and having 3–4 × 10^5^ relative light unit (RLU), and incubated at 37 °C for 2 h. After incubation, 100 μL of each mixture were introduced onto BHK-21 monolayers in a 96-well plate, with each sample tested in duplicates and incubated at 37 °C for a duration of 24 h. The bioluminescence-emitting cells were quantified using the Fluoroskan Ascent Luminometer (Thermo Fisher Scientific, MA, USA) and neutralization titers were then measured and recorded.

### 2.9. Coupling of Rabies M and Rabies G Proteins on Luminex Beads

Rabies Matrix (M) protein (abcam-ab236803) and Rabies G Protein (abcam-ab225593) were purchased from abcam. The rabies G and M recombinant proteins containing C-terminal his tag were coated on Luminex MagPlex-C Microspheres beads (M (bead region-34) and G (bead region-78)) as described in protocol 4.2.1. Carbodiimide coupling was present in xMAP^®^ Cookbook Luminex 5th edition [10]. In brief, 25 μg of each protein was coupled with luminex beads at 5 μg/1 × 106 beads. Presence of the proteins were confirmed by the anti-his antibodies (Cat no-AB72467) on Luminex beads.

### 2.10. Luminex Bead-Based Immune Assay

MagPlex beads coated with Rabies G and Rabies M antigens were used in this assay to detect antibodies present in the equine sera. The beads were first vortexed and sonicated then diluted in 1% bovine serum albumin (BSA). A total of 3 μL serum was added to the 147 μL of diluent (BSA) (1:50 dilution) to alternate columns of 96-well dilution plate. A total of 50 μL of MagPlex beads mixture was combined with 50 μL of diluted serum and incubated for 90 minutes on shaker at 800 rpm. To wash the MaxPlex beads, the plate was placed on a magnetic holder for 45 s, after which the supernatant was discarded, and 150 μL of fresh diluent was added and washing process was repeated two more times. Wells A1 and A2 served as blanks, while wells B1 and B2 contained anti-His-tagged antibody as the assay working control (100 μL per well). Wells C1 and C2 were filled with confirmed positive human sera as a positive control. Horse serum alone was added to wells D1 and D2, and the pseudovirus cminontrol was placed in wells E1 and E2. The remaining wells were used for diluted serum samples, added in duplicate. After this, the plate was incubated for 1 h with shaking at 800 rpm, followed by the three washes as previously described. Then, the plate was removed from the magnetic holder and the beads were resuspended in 75 μL of the diluent. The observation was made by using the Mag-pix Instrument (Luminex’s xMAP^®^ MAGPIX^®^, Austin, TX, USA).

### 2.11. Antibody Detection and Virus Neutralization Correlation

To determine the correlation between luciferase assay and the antibody detection assay, the serum dilution was performed by serially diluting it from 1:1600 to 1:3,276,800. The neutralization and Luminex assays were performed with the hyperimmunized equine serum samples #207, #217 and #220 in parallel and Spearman’s rank correlation coefficient (ρ) was calculated.

### 2.12. Statistical Analysis

The statistical analysis was performed using the GraphPad Prism 9.3.1 software. Data are presented as the mean ± SEM. Mean differences among groups were assessed for statistical significance employing student’s *t*-test and one-way ANOVA, followed by the Tukey test. For calculating the correlation, Spearman’s rank correlation coefficient (ρ) was used, given the non-parametric distribution of the data. Significance was denoted to *p*-values below 0.05, and denoted as follows: * for *p* < 0.05, ** for *p* < 0.01, *** for *p* < 0.001, and **** for *p* < 0.0001.

## 3. Results

### 3.1. Rabies-G Expression Analysis in HEK-293T Cells Post-Transfection

**Figure 2:**
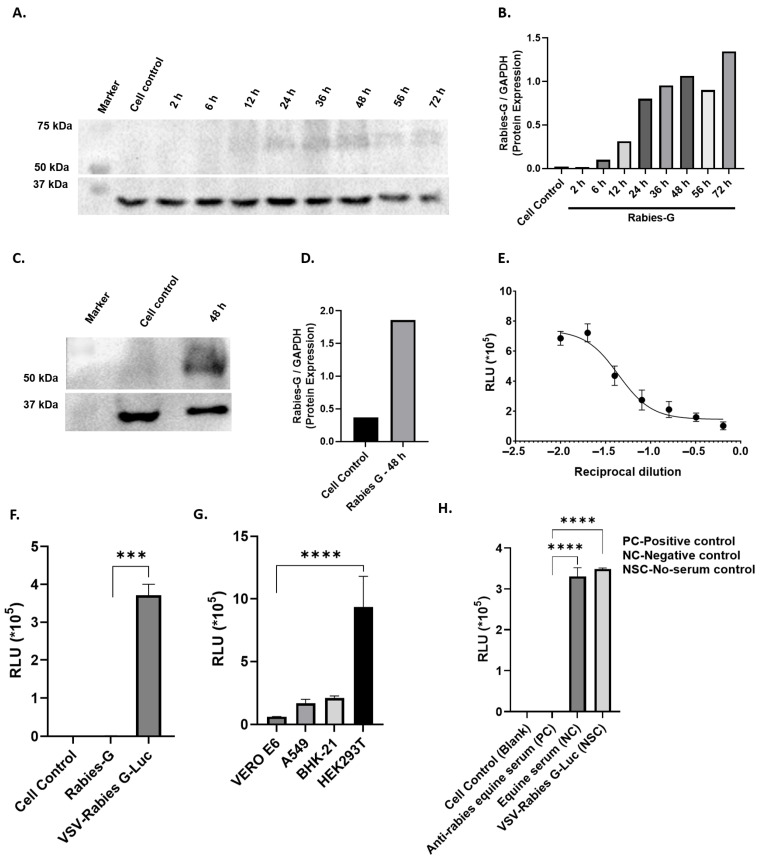
Generation and expression analysis of Rabies-G and VSV-Rabies-G-Luc pseudovirus. (**A**) Western blot showing Rabies-G protein expression in HEK293T cells at various time points post-transfection with Rabies-G pcDNA3.1 plasmid; GAPDH used as loading control. (**B**) Densitometric quantification of Rabies-G expression corresponding to panel A. (**C**) Western blot confirming Rabies-G expression in transfected HEK293T cells at 48 h and its incorporation into VSV-Rabies-G pseudovirus particles. (**D**) Quantification of Rabies-G band intensity from panel C. (**E**) Titration curve of VSV-Rabies-G-Luc pseudovirus in BHK-21 cells; *n* = 2. (**F**) Luciferase activity measured in BHK-21 cells infected with serial dilutions of VSV-Rabies-G-Luc pseudovirus to assess infectivity; *n* = 2. (**G**) Bar graph comparing luciferase expression across different cell lines (BHK-21, HEK293T, A549, and Vero E6) infected with VSV-Rabies-G-Luc pseudovirus; *n* = 2. (**H**) Neutralization assay using control equine serum and anti-rabies equine serum showing reduced luciferase signal, validating the pseudovirus-based neutralization platform; *n* = 2. The statistical data is represented as mean ± SEM *** *p* < 0.001, and **** *p* < 0.0001 when compared to respective group.

The schematic plan for generating rabies pseudovirus in HEK293T cells, expression analysis in various cells lines (Vero E6, A549 and BHK-21), and the development of a serological assay is shown in Figure 1. Briefly, HEK293T cells were transfected with an eukaryotic expression plasmid encoding the Rabies-G protein. In order to determine the Rabies-G protein expression post-transfection, culture supernatant samples were collected at 2 h, 6 h, 12 h, 24 h, 48 h, and 72 h. Immunoblotting analysis showed peak expression of the Rabies G protein in HEK293T cells between 48 and 72 h post-transfection, as shown in Figure 2A,B, which was further confirmed in the transfected HEK293T cells at 48 h (Figure 2C,D).

### 3.2. Generation of Rabies-G VSV-Luc Pseudovirus and Infection Analysis Across Cell Lines

After confirming peak Rabies-G expression at 48 h post-transfection, the cells were infected with ΔG VSV-Luc pseudovirus, and the resulting VSV-Rabies-G-Luc pseudovirus was harvested 24 h later. The VSV-Rabies-G-Luc pseudovirus was titrated to determine the optimal volume for testing by performing two-fold serial dilutions ranging from 100 µL to 1.5625 µL in BHK-21 cells. It was observed that a pseudovirus volume of approximately 6.25 µL produced 50% of the maximum luciferase signal, making it the chosen volume for subsequent experiments, as shown in Figure 2E. In parallel, we assessed the luciferase activity in VSV-Rabies-G-Luc-transfected cells in comparison to Rabies-G-pcDNA3.1-transfected cells (Figure 2F), confirming successful Rabies-G-Luc transfection. Furthermore, we determined the luciferase activity of the VSV-Rabies-G-Luc pseudovirus in different cell lines, i.e., HEK293T, BHK2l, VERO E6, A549 through bioluminescent luciferase activity analysis as shown in Figure 2G. Among all the four cell lines tested, high activity was detected in both HEK293T and BHK21 in cell lines. However, BHK-21 was chosen for further experimentation due to greater convenience and stable luciferase activity compared to HEK293T.

### 3.3. Evaluation of Neutralization of VSV-Rabies-G-Luc Pseudovirus by Anti-Rabies Equine Serum

Luciferase expression of the VSV-Rabies-G-Luc pseudovirus system is tested with control equine serum samples and positive anti-rabies equine serum as shown in Figure 2H. The data confirms the effectiveness of the pseudovirus system, as evident from the clear distinction in luciferase activity between the control and positive serum samples.

### 3.4. Optimization of Equine Serum Dilution for the Serum Neutralization Test

**Figure 3:**
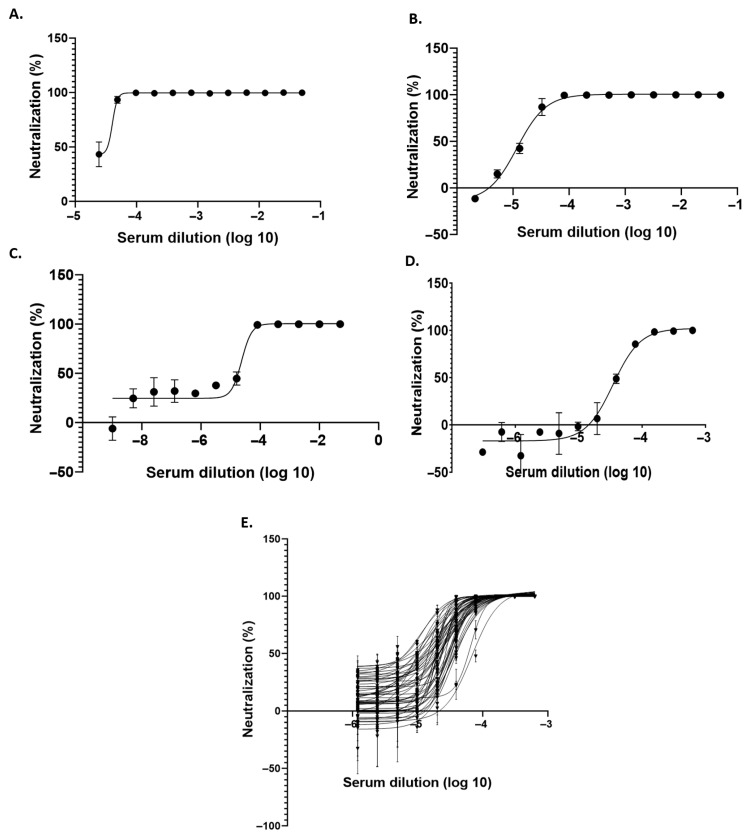
Neutralization assay of equine serum samples using VSV-Rabies-G-Luc pseudovirus. (**A**–**D**) Luciferase-based neutralization curves showing VSV-Rabies-G-Luc pseudovirus infection in BHK-21 cells incubated with serially diluted equine serum samples at different dilution schemes: (**A**) two-fold dilutions starting from 1:40, (**B**) 2.5-fold dilutions from 1:40, (**C**) five-fold dilutions from 1:40, and (**D**) two-fold dilutions starting from 1:1600. (**E**) Screening of equine serum panel using the neutralization assay in BHK-21 cells to assess the presence of rabies-specific neutralizing antibodies based on luciferase signal reduction; *n* = 62. All serum dilutions are represented on an log 10 scale.

The reproducibility of pseudovirus neutralization assay depends on using the right combination of pseudovirus particles and serum dilution. In order to determine the optimum serum dilution to be used for neutralization assays, we screened three different types of serial dilution of serum (two fold, 2.5 fold, and five fold) for assessing the neutralization titer against the VSV-Rabies-G-Luc pseudovirus using luciferase assay, as illustrated in Figure 3A–C. Among these, reproducible results were observed using the two-fold dilution but not the others. Subsequently, we conducted the neutralization assay using Rabies G pseudovirus with an initial serum dilution of 1:1600 (Figure 3D), which was determined to be the most effective assay protocol.

### 3.5. Serum Neutralization Test

In order to confirm the suitability of the VSV-Rabies-G-Luc pseudovirus as a substitute for live RV in a serum neutralization assay, neutralization assays were performed using serum sample from horses either naïve or immunized with RV. The results showed that pre-incubation with the equine sera neutralized the infectivity of VSV-Rabies G-Luc pseudovirus in a dose-dependent manner as shown in Figure 3E. No neutralization was observed with sera obtained from the horse which has been considered as negative control for the experiment. The results confirmed the presence of anti-Rabies-G antibodies in the equine serum samples and validated the efficacy of the VSV-Rabies-G-Luc pseudovirus system. Using these findings, ND_50_ and IC_50_ values were calculated, as presented in Supplementary Table S1.

### 3.6. Antibody (IgG) Response in Anti-Rabies Equine Serum Samples

**Figure 4:**
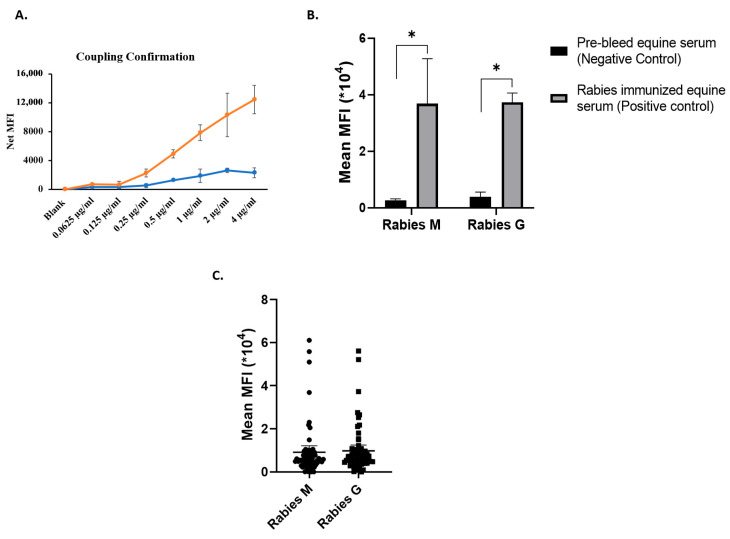
Luminex bead-based immunoassay for detection of rabies-specific antibodies. (**A**) Confirmation of successful coupling of Rabies M and G proteins to Luminex beads using anti-6XHis antibodies. (**B**) Assay validation using control (*n* = 10) and anti-rabies equine serum (*n* = 3), demonstrating specific antibody binding to the coupled beads. (**C**) Screening of equine serum samples (*n* = 52) for antigen-specific IgG responses against Rabies M and G proteins using the bead-based Luminex platform, expressed as median fluorescence intensity (MFI). The statistical data is represented as mean ± SEM. * *p* < 0.05 when compared to respective group.

In spite of the ease of use when compared to live RV, use of the pseudovirus system is labor- and time-intensive. Correlation of neutralization titre with an antibody binding assay that can be performed faster will be helpful in providing faster results. For this purpose, we also developed a Luminex-based assay for quantitative estimation of antibody titer in serum samples. Briefly, purified antigen corresponding to Rabies M and Rabies G proteins were conjugated to Luminex beads as described in Mani et al. 2018 [11]. The coupled proteins were tested and confirmed by the Luminex assay using anti-His antibody as shown Figure 4A. We proceeded to test for the presence of anti-rabies G and M antibodies in rabies-immunized equine serum; using positive control equine serum samples, as demonstrated in Figure 4B, we observed specific antibody response against M and G antigens in anti-rabies equine serum compared to equine serum. We further screened all the equine samples to detect antibody response. As shown in Figure 4C, all the hyperimmunized samples showed antibody response in the samples as compared to the naive equine samples, demonstrating the specificity of the assay.

### 3.7. Correlation of the Antibody Response in Neutralization Assay and Luminex-Bead-Based Analysis

**Figure 5:**
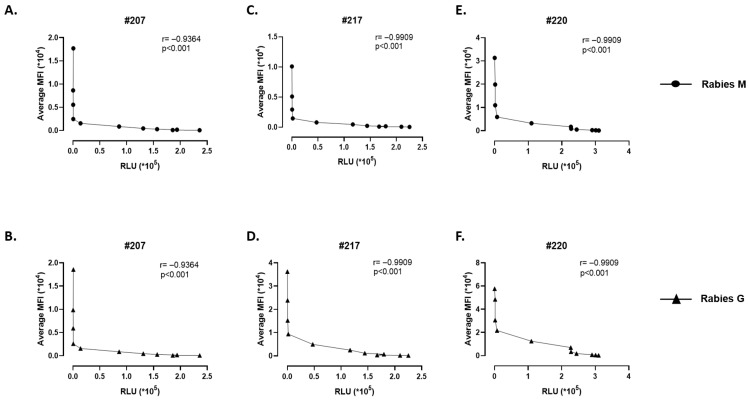
Correlation between binding antibody against Rabies G and M measured by Luminex assay and VSV-Rabies G-Luciferase pseudovirus neutralizing activity. (**A**–**F**) The figure depicts the correlation between antigen-specific IgG responses against Rabies-M (**A**–**C**) and Rabies-G (**D**–**F**) in comparison to neutralizing antibody activity in serum samples (sample-207, 217 and 220); *n* = 2. IgG levels against Rabies-M and Rabies-G antigen were quantified using a Luminex bead-based multiplex assay and expressed as median fluorescence intensity (MFI). Neutralizing antibody titers were determined using a VSV-Rabies-G pseudovirus expressing a luciferase reporter. Luciferase activity, measured 24 h post-infection, served as an inverse proxy for neutralization. Spearman’s rank correlation coefficient (ρ) was calculated between Luminex MFI and Luciferase readings along with two-tailed *p*-value using GraphPad Prism. Each data point represents an individual serum sample tested in parallel in both assays.

We determined the correlation of antibody response in the equine serum sample through two developed antibody assays, which are the pseudovirus-based neutralization assay and Luminex-bead based assay. We tested three equine serum samples and plotted the antibody response and the neutralization response with different serum dilution. As shown in Figure 5A–E, it was observed that the correlation coefficient R was (−0.9364 (sample 207), 0.9909 (sample 217), and 0.9909 (sample 220)) with the *p* value <0.001.

## 4. Discussion

RV, a fatal zoonotic pathogen, requires high-containment facilities for live virus research, which restricts large-scale serological and vaccine evaluation. To overcome these biosafety limitations, pseudovirus-based systems offer a safer and similarly quantitative alternative for the determination of rabies-specific neutralizing antibody titers under BSL-2 conditions. The present study presents data with respect to the establishment and characterization of a VSV–Rabies-G-Luc pseudovirus system along with an in-house Luminex bead-based diagnostic platform to evaluate rabies-specific immune responses. To generate the pseudovirus platform, HEK293T cells were transfected with a plasmid encoding the Rabies-G glycoprotein, resulting in robust protein expression at 48 h post-transfection, confirmed by Western blot analysis, at which point the cells were infected with VSV-ΔG-Luc to produce the VSV–Rabies-G–Luc pseudovirus. The generated VSV–Rabies-G–Luc pseudovirus exhibited strong luciferase activity upon infection in both HEK293T and BHK-21 cells, validating its functionality and BHK-21 cells were further chosen for their stable and consistent luciferase expression, as well as convenience of use. Moreover, neutralization assays using the anti-rabies equine serum against the pseudovirus demonstrated the system’s potential as an effective diagnostic tool. In parallel, an in-house Luminex bead-based multiplex assay was developed to detect antibodies against the Rabies-G and Rabies-M proteins. The binding antibody levels measured by this platform showed a strong correlation with neutralization titers observed in hyperimmunized equine sera, validating its reliability for serological assessment. Overall, this study establishes a robust and effective VSV–Rabies-G pseudovirus platform, complemented by an in-house Luminex-based antibody detection assay, providing a safe and scalable BSL-2-compatible system for comprehensive evaluation of rabies-specific immunogenicity and neutralization responses, which can serve as a valuable diagnostic and research tool to accelerate vaccine development against the highly fatal rabies virus.

Rabies is endemic in India, with the highest burden in rural areas due to high stray dog populations and limited healthcare access. India accounts for 36% of global rabies deaths, with over 20,000 annual fatalities. Domestic dogs are the primary reservoirs, responsible for over 90% of cases, while other animals play a lesser role. Key factors driving rabies incidence include stray dog populations, poor healthcare access, low public awareness, and inadequate animal control measures [1,12]. India’s high burden of the disease calls for live virus research in BSL-3/4 labs, highlighting the need for advanced tools adaptable to BSL-2 to reduce rabies fatalities [13]. Therefore, pseudoviruses offer a safer alternative to live attenuated viruses in diagnostics, as they lack the pathological infectious proteins while still responding to neutralizing antibodies against viral structural proteins in a manner similar to live virus, making them promising vaccine candidates and diagnostic tools. For instance, vaccine studies using SARS-CoV-2 S protein-bearing pseudo-typed viruses demonstrated higher sensitivity compared to conventional neutralization assays with live SARS-CoV-2, highlighting their effectiveness in eliciting immune responses [14]. Similarly, pseudo-typed viruses incorporating the Rabies-G protein have been developed to study viral entry mechanisms, but their potential for diagnostic applications remains unexplored [15].

Rabies continues to pose a major public health challenge in India, accounting for several thousand human deaths each year. The country currently employs a range of modern cell culture-based vaccines, such as the purified vero cell rabies vaccine (PVRV) and purified chick embryo cell vaccine (PCECV) [16,17], as well as the recently introduced recombinant nanoparticle-based vaccine (ThRabis) [18], which offer superior safety and immunogenicity compared to the older nerve tissue vaccines. In addition, rabies immunoglobulins are administered as part of both pre-exposure (PrEP) and post-exposure prophylaxis (PEP) regimens to provide immediate passive immunity following potential exposure. National initiatives such as the National Rabies Control Program (NRCP) further strengthen the country’s response by improving access to vaccines and immunoglobulins, promoting mass dog vaccination using modified live virus (MLV) vaccines, and raising public awareness on timely wound management and PEP adherence to prevent rabies fatalities. However, significant challenges persist, including low vaccine coverage, managing the large stray dog population, and inadequate healthcare infrastructure in rural areas.

A major obstacle in advancing rabies research and vaccine development is the limited availability of BSL-3/BSL-4 facilities required for handling live rabies virus safely. This restricts the scope of studying viral mechanisms and testing vaccine candidates. The current study on VSV-Rabies-G pseudovirus establishment offers a promising alternative by providing a system that mimics the rabies virus. This approach allows researchers to study viral entry, test vaccine efficacy, and screen for neutralizing antibodies without the need for high-containment facilities. By enabling safer and more accessible research, pseudovirus systems can significantly contribute to accelerating vaccine development and improving rabies control strategies in India.

## 5. Conclusions

Rabies remains a major public health concern in India, but significant progress has been made in vaccine development and control strategies. Addressing the challenges associated with vaccine coverage, stray dog management, and healthcare infrastructure is crucial for further reducing the incidence of rabies. Continued research, public awareness, and comprehensive control programs are essential to achieving the goal of rabies elimination. To overcome these gaps, we have established a robust serological assay for detecting rabies-specific antibodies using a multiplex Luminex and a pseudovirus-specific neutralization assay in BSL-2 setup, which provides an edge of the BSL3 facility and helps us to evaluate the efficacy of the developing antiviral and vaccine candidates against rabies in India.

## Data Availability

All the data generated and anlysed is included in the manuscript and Supplementary Material. Further data that supports the findings of this study are available from the corresponding author upon reasonable request.

## Supplementary Materials

The following supporting information can be downloaded at: https://www.mdpi.com/article/10.3390/v18010108/s1, Table S1: Showing the Inhibitory concentration (IC50) of Pseudo-virus (VSV-Rabies-Luc) and Neutralization doses (ND50) of Antibodies present in the serum samples (N=51).
