# Supplementary material for: Serological Assays to Measure Rabies Antibody Response in Equine Serum Samples
Source: Viruses. 2026 Jan 14;18(1):108. doi: 10.3390/v18010108 (PMC12846700; doi:10.3390/v18010108)
Supplement: Supplementary file 1 [file viruses-18-00108-s001.zip › viruses-4001507-supplementary.pdf]

Table 1: Showing the Inhibitory concentration (IC50) of Pseudo-virus (VSV-Rabies-Luc) and Neutralization doses (ND50) of Antibodies present in the serum samples (N=51).

Linear Regression Analysis:

Sr. No.	Sample ID	IC50	ND50	Sr. No.	Sample ID	IC50	ND50
**1**	**S-20**	4.09E-05	24479.8	**16**	**S-259**	1.79E-05	55897.15
**2**	**S-30**	1.93E-05	51706.31	**17**	**S-283**	2.08E-05	48123.2
**3**	**S-31**	3.68E-05	27144.41	**18**	**S-288**	2.7E-05	37050.76
**4**	**S-4**	2.02E-05	49407.11	**19**	**S-295**	2.93E-05	34118.05
**5**	**S-45**	1.63E-05	61387.35	**20**	**S-313**	2.28E-05	43821.21
**6**	**S-71**	1.39E-05	72046.11	**21**	**S-318**	3.37E-05	29638.41
**7**	**S-79**	3.66E-05	27300.03	**22**	**S-319**	3.02E-05	33134.53
**8**	**S-92**	1.16E-05	86355.79	**23**	**S-362**	1.94E-05	51626.23
**9**	**S-106**	1.68E-05	59665.87	**24**	**S-363**	7.31E-05	13676.15
**10**	**S-110**	3.95E-05	25342.12	**25**	**S-384**	2.69E-05	37216.23
**11**	**S-158**	1.77E-05	56369.79	**26**	**S-479**	5.66E-05	17683.47
**12**	**S-178**	1.12E-05	89047.2	**27**	**S-484**	1.44E-05	69541.03
**13**	**S-186**	2.88E-05	34698.13	**28**	**S-491**	1.86E-05	53676.87
**14**	**S-207**	7.31E-06	136892.5	**29**	**S-337**	1.92E-05	52219.32
**15**	**S-217**	9.46E-06	105753	**30**	**S-342**	8.97E-06	111457.9

**31**	**S-220**	3.2E-05	31240.24	**42**	**S-347**	6.44E-06	155351.9
**32**	**S-231**	1.34E-05	74682.6	**43**	**S-403**	8.61E-06	116157.5
**33**	**S-232**	9.66E-06	103519.7	**44**	**S-414**	6.34E-06	157703.8
**34**	**S-237**	2.79E-05	35803.8	**45**	**S-415**	3.32E-06	301295.6
**35**	**S-242**	6.48E-06	154344.8	**46**	**S-418**	1.81E-05	55218.11
**36**	**S-245**	9.13E-06	109517	**47**	**S-426**	2.51E-06	397772.5
**37**	**S-246**	2.33E-05	42992.26	**48**	**S-435**	4.66E-06	214822.8
**38**	**S-248**	1.01E-05	98911.97	**49**	**S-437**	8.61E-06	116157.5
**39**	**S-252**	1.76E-05	56915.2	**50**	**S-455**	6.34E-06	157703.8
**40**	**S-253**	1.43E-05	69783.67	**51**	**S-467**	3.32E-06	301295.6
**41**	**S-257**	1.21E-05	82712.99				
